# Implementation of the CDC translational informatics platform - from genetic variants to the national Swedish Rheumatology Quality Register

**DOI:** 10.1186/1479-5876-11-85

**Published:** 2013-04-02

**Authors:** Imad Abugessaisa, David Gomez-Cabrero, Omri Snir, Staffan Lindblad, Lars Klareskog, Vivianne Malmström, Jesper Tegnér

**Affiliations:** 1 Department of Medicine, The Unit of Computational Medicine, Center for Molecular Medicine, Karolinska Institutet, Solna, Sweden; 2 Department of Medicine, The Rheumatology Unit, Center for Molecular Medicine, Karolinska Institutet, Solna, Sweden; 3 Rheumatology clinic, Karolinska University Hospital, Solna, Sweden; 4 Current affiliation: Department of Immunology, Centre for Immune Regulation, Oslo University Hospital-Rikshospitalet, University of Oslo, Oslo, Norway

**Keywords:** Swedish Rheumatology Quality Register (SRQ), Translational medicine platform, Secondary use of clinical data, Patient de-identification

## Abstract

**Background:**

Sequencing of the human genome and the subsequent analyses have produced immense volumes of data. The technological advances have opened new windows into genomics beyond the DNA sequence. In parallel, clinical practice generate large amounts of data. This represents an underused data source that has much greater potential in translational research than is currently realized. This research aims at implementing a translational medicine informatics platform to integrate clinical data (disease diagnosis, diseases activity and treatment) of Rheumatoid Arthritis (RA) patients from Karolinska University Hospital and their research database (biobanks, genotype variants and serology) at the Center for Molecular Medicine, Karolinska Institutet.

**Methods:**

Requirements engineering methods were utilized to identify user requirements. Unified Modeling Language and data modeling methods were used to model the universe of discourse and data sources. Oracle11g were used as the database management system, and the clinical development center (CDC) was used as the application interface. Patient data were anonymized, and we employed authorization and security methods to protect the system.

**Results:**

We developed a user requirement matrix, which provided a framework for evaluating three translation informatics systems. The implementation of the CDC successfully integrated biological research database (15172 DNA, serum and synovial samples, 1436 cell samples and 65 SNPs per patient) and clinical database (5652 clinical visit) for the cohort of 379 patients presents three profiles. Basic functionalities provided by the translational medicine platform are research data management, development of bioinformatics workflow and analysis, sub-cohort selection, and re-use of clinical data in research settings. Finally, the system allowed researchers to extract subsets of attributes from cohorts according to specific biological, clinical, or statistical features.

**Conclusions:**

Research and clinical database integration is a real challenge and a road-block in translational research. Through this research we addressed the challenges and demonstrated the usefulness of CDC. We adhered to ethical regulations pertaining to patient data, and we determined that the existing software solutions cannot meet the translational research needs at hand. We used RA as a test case since we have ample data on active and longitudinal cohort.

## Background

As stated above, the development of genomic technologies following the post-genomic era have resulted in immense large-scale of data generation. However, it is not only large volumes of DNA sequence data generated from large-scale projects, e.g., (e.g. 1000 genome [1] and ENCODE [2]), that pose challenges for computing infrastructures. Technological advances have opened new windows into genomics beyond the DNA sequence. Some examples of different types of data that can be generated today—from inside the cell—include: DNA-methylations, SNPs, CNVs, protein coding RNA, non-coding RNA, splice variants, histone modifications, nucleosome positions, transcription factors and their DNA binding sites, transcription start-sites, promoters, protein-protein interactions, protein localization, protein modifications (these are numerous), DNA binding proteins, and metabolites. In addition clinical practice and healthcare have produced large amounts of data describing diseases, medications, environmental factors, and lifestyle-related information. Clinical data stored in electronic medical records is very restricted and managed differently than research data commonly shared and available through public repositories or scientific journals. The lack of appropriate and useable computing infrastructure reduces the utilization of data sources, which have much greater research potential than is currently realized. In particular, there is an urgent need for computing resources to connect both molecular and healthcare data. Current challenges to be addressed are secure and easy access to biomedical databases, patient data protection, data sharing, and database integration. The current lack of methods and systems to bridge the gap between research and clinic information constitute a major road-block for translational research and for the benefit of healthcare.

The current research addresses the above challenges and provides an informatics platform for modeling and integrating multiple data sources in the Rheumatology Research Laboratory at the Center for Molecular Medicine (CMM), Karolinska Institute (KI) and the clinical data at the Rheumatology Clinic at Karolinska University Hospital in the other hand. The data sources at CMM are the rheumatoid arthritis (RA) biobank (serum, EDTA-plasma and DNA), cell registry (PBMC, SFMC, etc.), genotype variants, and serology database for a cohort of 379 patients diagnosed with RA (defined by ACR 1987 or later ACR/Eular 2010).

The cohort presents three profiles:

HLA-DR genotyping.

Genotype of 65 SNPS all predisposing for RA either directly or in interaction with HLA.

Detection of anti-CCP antibodies IgG antibodies against citrullinated alpha-enclose peptide-1 (CEP-1) and citrullinated type-II collagen (citC1III), IgG antibodies against citrullinated vitamin.

At the Rheumatology Clinic, data about disease duration, treatment, disease activity, and specification of the disease are stored in the SRQ [3]. A translational medicine platform that integrates all data sources is the key to making research even more translational [4], and it will also empower current research to find predictive markers, such as immunological phenotypes.

### Rheumatoid arthritis

RA is a common chronic inflammatory debilitating disease that primarily affects the synovial joints, but it may also affect tissues and organs. For patients, quality of life and the possibility of maintaining employment is significantly affected. The life time risk of developing RA in Sweden is around 2% [5], and despite the use of the new improved therapies, the rate of sick leave in early RA is still close to 50% [6]. Risk factors for developing RA have been mapped to both genetic and environmental factors, with the Human Leukocyte Antigen (HLA) region and cigarette smoking conferring the strongest risk [7]. The HLA association is tightly linked to the emergence of a set of autoantibodies denoted as ACPA (anti-citrullinated protein antibodies) [8], which today are used to subcategorize this disease. Hence, immunological studies aimed at increasing our understanding of disease initiation and perpetuation needs to take into account both the genetic and serological profile of the included patient material.

### i2b2 and STRIDE: community driven software solutions

A number of technology platform solutions are available to manage biomedical data in translational research. Some of them, developed by research community are released as open-source under General Public License (GPL [9]), developed by research communities at universities and research institutes. One of the commonly used platforms is Informatics for Integrating Biology and the Bedside (i2b2) [10]. The i2b2 platform is funded by the National Institutes of Health (NIH). i2b2 uses The International Classification of Diseases (ICD) [11] as a taxonomic standard to classify diseases, and it enables the creation of formal ontologies to meet the specific requirements of different research studies.

The design of i2b2 provides software platform and scalable solutions that facilitate repurposing of clinical data into the research setting and to secure the access and management of patient information for research purposes. i2b2 was implemented as a set of software cells orchestrated in hive architecture that communicate via web service technology in a Service-Oriented Architecture (SOA) environment. This kind of architecture provides secure communication based on Simple Object Access Protocol (SOAP) messages. The principle design of i2b2 paid attention to query and data retrieval performance. Two predefined test cases were supported by i2b2, as mentioned in [12]:

1. Explore patient data to find sets of patients that would be of interest for further research, and

2. Make use of the detailed data provided by the Electronic Medical Record (EMR) to discover different phenotypes of the set of patients identified (first test case) in support of genomic, outcome, and environmental research.

Based on the Health Level 7 (HL7) data model, the Stanford Translational Research Integrated Database Environment (STRIDE) represents an integrated standards-based translational research informatics platform. It provides a number of functionalities required in translational research [13]. The basic building blocks of STRIDE are; a clinical data warehouse based on the Health Level Seven (HL7) Reference Information Model (RIM) [14], an application development framework for building research data management applications on the STRIDE platform and a biospecimen data management system.

In addition to the EMR, STRIDE provides biobank data management. Similar to i2b2, STRIDE uses ICD and other standards like Systematized Nomenclature Of Medicine Clinical Terms (SNOMED) [15] to build the semantic model to represent biomedical concepts and different types of relationships. The data warehouse of STRIDE built on Oracle 11g, the database organized in three logically clustered databases; clinical data warehouse, research data management and biobanks. The schemas used based on an Entity-Attribute-Value (EAV) model and object-oriented data structures derived from the HL7. Different software components of STRIDE are communicating via set of web services in a service oriented architecture (SOA) platform. Through the semantic layer, STRIDE support standards-based data entry, data integration, data retrieval and data interoperability.

### Translational informatics challenges and solutions

Due to the different storage strategies for patient data and the explosion in volume, translational informatics faces a significant challenge in database integration. At the research level, the increasing pace of molecular data-production through high throughput technologies creates a great demand for data management (storage, transfer, retrieval, processing, and interpretation). On the other hand, patient data at the health care level is becoming more complicated since patient records are stored in EMR and the quality of care registry for different diseases. Re-use of clinical data in the research setting brings data management challenges. Data management includes not only storage of the data, but also access restrictions and control. Researchers need to perform queries across different data sources (patient bio samples, genetics, serology, etc.) and clinical data (diagnosis, medications, diseases activities, life style) from healthcare facilities. Our approach is to collect and define end-user requirements (biomedical and bioinformatics researchers) for the study the etiology, pathogenesis, disease course, co-morbidities, and therapies of RA. We matched the requirements with the current solutions and used engineering methods to implement the system at the CMM. By selecting and implementing the CDC from Oracle™ (see the method sections), we achieved our objectives and satisfied end-user requirements.

## Methods

Systematic research methods are essential to (1) determine the services and functionality to be provided by the system, (2) identify and understand the operational constraints (patient privacy, security, etc.), and (3) understand bioinformatics workflow management and analysis.In the following sections, we discuss different methods and techniques used during the current study and present the results from each method.

### Universe of discourse (UoD)

We started the research by identifying sources of data and acquiring a knowledge base. In such a domain, all entities (objects) composing the domain and the relationship among them were identified. This domain of knowledge is known as the Universe of Discourse (UoD) [16], ‘a complete range of objects, events, attributes, relations, ideas, etc. that are assumed to exist at one occasion’. In a database management system, UoD refers to the part of the world under study; the UoD maps all relevant aspects of the investigated world. Its conceptualization (abstraction) must be complete and comprehensive. To define the UoD, we used two tools: the first was the Unified Modeling Language (UML) class diagram, which shows all classes and their relationships; secondly concept mapping, a technique to define concepts and their attributes.

### Class diagram

**Figure 1:**
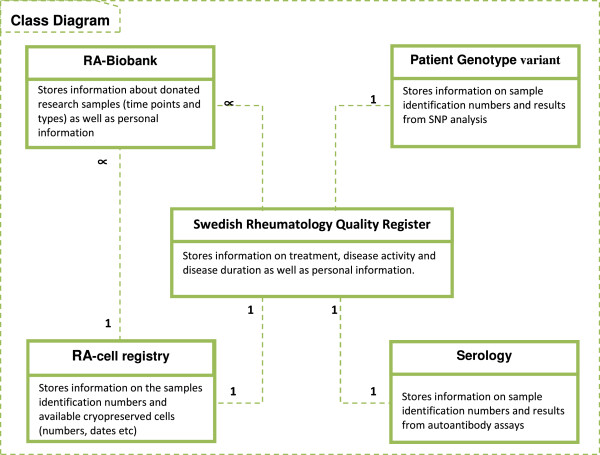
A class diagram for the translational medicine computing platform for RA.

The class diagram is a UML artifact [17]. UML is a modeling approach system development (object oriented design and analysis). UML was used to diagrammatically visualize and document software modules during requirements analysis. UML provides three types of diagrams: behavior, interaction, and structure diagrams ([18] and [19]). The class diagram is one of the structure diagram types and was used to model the basic elements of the software system, their attributes, and relations. The class diagram in Figure 1 illustrates the main elements of the UoD under study. At the heart of the class diagram is the Swedish Rheumatology Quality Register, in which RA patient records are created and stored. Each patient was defined with a set of personal health information (PHI); the two important attributes were the civic registration number and the patient code at the registry, which is unique to each patient.

The other classes are the RA-Biobank, serology, genotype variant, and the cell registry.

The first three classes used the civic registration number as the primary attribute to define patient samples, and the secondary key attribute is the SYF number, which is a project number and is unique to each patient. The fourth class (cell registry) only uses the SYF number as a key attributes. The relationship between all classes is specified in the diagram.

### Common vocabulary from UML class diagram

To overcome definitional and semantic problems, we built a common vocabulary that helps to identify the concepts (attributes) used in the class diagram. There are different ways to model the common vocabulary for a particular domain, including ontology engineering methods (OEM). OEM is used to give a formal explicit description of concepts in UoD [20]. Creating domain ontology is beyond the scope of this research since we are not going to apply any annotation or semantic web techniques. Here, we used concepts from OEM to create the common vocabulary for the translation medicine computing platform using knowledge acquisition and representation tools.

There is no one “correct” way for developing common vocabulary [21]. We used the conceptual map [22] to represent and communicate knowledge between biomedical researchers, the lab technician, and the system developer. The common vocabulary consists of a set of classes. Each class (database source) has its attributes and properties. The common vocabulary for biobanks, Swedish Rheumatology Quality Register (SRQ) and the cell register derived from the current representation of each of the three sources. It should be mentioned that the SRQ (clinical database) do not confirm to any of the available clinical diagnosis standards instead using a pre-defined common RA diagnosis. To model the serology class we used the same output format and naming of the attributes provided by the Flow cytometry system. Finally, for the SNPs class , we used Human Genome Variation Society (HGVS) nomenclature to name the RS number for each variant [23], see Table 1.

**Table 1:** List of selected SNPs

		**Allele frequencies**		
Rs number	**Locus**	**Major**	**Minor**	**Risk**
6314	HTR2A	C	T	C
1328674	HTR2A	C	T	T
548234	PRDM1	T	C	C
4781003	CIITA	C	T	T
4535211	PLCL2	G	A	A
10431908	CIITA	A	G	G
544167	C2	G	T	G
12746613	FCGR2A	C	T	T
4810485	CD40	G	T	G
10498441	NID2	A	G	A
10499194	OLIG3,TNFAIP3	C	T	C
2064476	HLA-DPB2	A	G	A
706778	IL2RA	C	T	T
2736340	BLK	A	G	G
26232	C5orf30	C	T	C
540386	TRAF6	C	T	C
231707	C4orf8	G	A	A
10402677	CEACAM1	G	A	A
42041	CDK6	C	G	G
2024301	CLEC4A;POU5F1P3	A	T	T
3807306	IRF5	A	C	A
10488631	IRF5;TNPO3	T	C	C
3761847	TRAF1/C5	A	G	G
7026551	C5	A	C	C
11586238	CD2,CD58	C	G	G
231735	CTLA4	G	T	G
13017599	REL	A	G	G
394581	TAGAP	T	C	T
2263484	C21orf74	A	C	C
6682654	CD244			
6859219	ANKRD55	C	A	C
13031237	REL	A	C	C
934734	SPRED2	A	G	G
11676922	AFF3	A	T	T
3087243	CTLA4	G	A	G
1678542	KIF5A	C	G	C
951500	CCL21	A	G	A
892188	GLP-1;FDX1L;ICAM5	C	T	T
1133104	CLEC4A;POU5F1P3	G	T	T
1980422	CD28	T	C	C
1859341	CEACAM8	A	G	G
3087456	CIITA	A	G	G
2271077	GALNTL2	A	G	A
2377422	CLEC4A;POU5F1P3	C	T	T
2476601	PTPN22	C	T	T
2812378	CCL21;C9orf144B	A	G	G
2240340	PADI4	C	T	T
6416647	CIITA	T	C	C
3890745	MMEL1	T	C	T
4272626	NHLH2	C	T	T
10258735	RPA3	A	G	G
3093023	CCR6	G	A	A
3218253	IL2RB	G	A	A
6822844	IL2,IL21	G	T	G
7234029	PTPN2	A	G	G
6457620	HLA-DRA	G	C	G
6920220	OLIG3,TNFAIP3	G	A	A
10413014	CEACAM8	A	G	G
7574865	STAT4	G	T	T
10468473	MAP2K4	G	A	A
10410147	CEACAM8	G	A	A
10919563	PTPRC	G	A	G
4750316	DKFZp667F0711/PRKCQ	G	C	G
2523451	MICA	A	G	G
6457617	HLA-DQ	C	T	C

### RA biobank and cell registry

The RA biobank and cell registry were created at the CMM and have been running and serving the purpose. The biobank stores and manages biological specimens from patients and from relevant individuals in the population. Biological specimens are available mainly in the context of specific research of one or several diseases (RA and MS (multiple sclerosis)), where these studies are conducted by one principal investigator or, in networks of clinicians and biomedical scientists. The biobank database has been constructed over specimen banks containing DNA, RNA, serum, plasma and several other preparations and management facilities, such as sample volume and box number.

**Figure 2:**
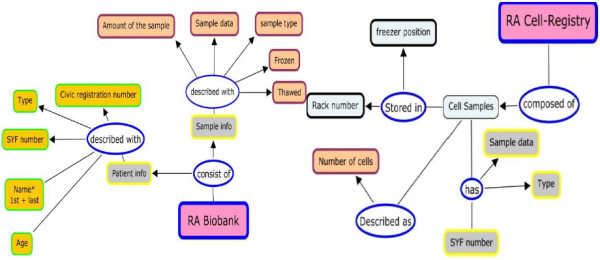
RA Biobank and the cell registry attributes.

For the cohort of the 379 patients, the biobank stores more than 15000 samples, which are available for researchers to run different studies. The current biobank system was implemented in Filemaker Pro a client/server system [24]. Filemaker Pro is a relational database system for handling and managing small enterprise database. It gives the user the flexibility to rapidly develop database schemas without any consideration to integrity constrains and database normalization. Filemaker Pro has limited capabilities to integrate different database sources and scale-up. A complementary source for the biobank is the cell registry, which is an excel sheet maintained manually, and it points to the location of cryopreserved cell samples in the freezer and racks. Moreover, it also gives quantitative information about the samples, such number of cells available. Both the RA-biobank and the cell registry vocabulary and attributes are illustrated in Figure 2.

### Genotype variants and serology

Genotype variants and serology datasets contain information derived from the biobanked specimens, and these databases contain extensive information concerning the following: genotypes, both HLA-type and extensive SNP data, and serology covering titers of different RA-related autoantibodies (both IgG and IgA), such as CCP, MCV, citrullinated enolase, vimentin, fibrinogen, and type II collagen [25]. These databases were maintained and managed by the researchers.

DNA was extracted from EDTA blood by the salting-out method [26]. Genotyping for HLA-DRB1 haplotypes was conducted using the sequence-specific primer-polymerase chain reaction method (DR low-resolution kit; Olerup SSP, Saltsjöbaden, Sweden). DRB1*04 and DRB1*01 positive patients were further subtyped by Olerup SSP DRB1*04 (Olerup SSP, Saltsjöbaden, Sweden) subtyping kits, respectively.

Genotyping for selected SNPs was performed using a 64-OpenArray platform (Applied Biosystems, Foster City, California, USA) using chip-based TaqMan genotyping technology. Genotyping was performed according to manufacturer’s instructions, and genotype call were made using AutoCaller (Applied Biosystems).

**Figure 3:**
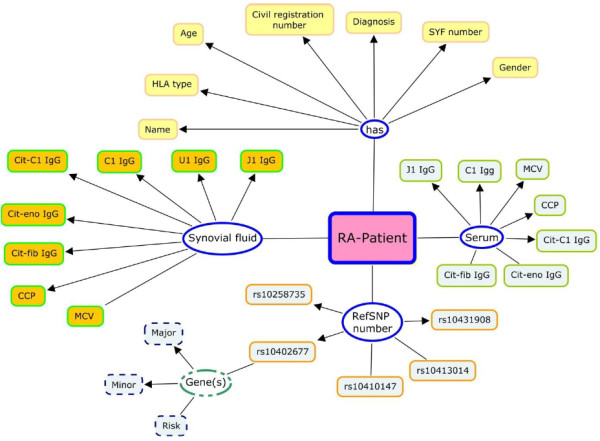
Illustration of Genotype and serology vocabulary.

Similar to the cell registry, this database was maintained in an excel spread sheet and has not been linked to any other sources. Patients were the main object in this database, and for each patient, different measures from synovial fluid to serum were stored. In addition, a list of references of SNP numbers were stored and categorized per gene risk level (Risk, Major, Minor). An illustration of the genotype and serology vocabularies is in Figure 3. The details of the selected SNPs are listed in Table 1.

### The swedish rheumatology quality registry

**Figure 4:**
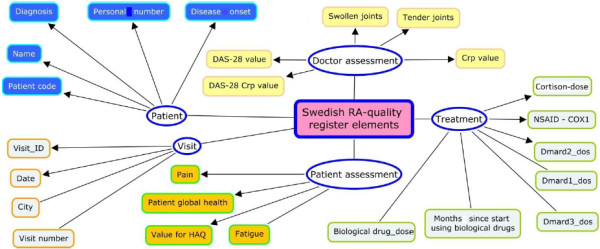
**The Swedish Rheumatology quality register.** Entities, attributes, and relationships. Dmard: Disease-modifying Anti-rheumatic Drugs. HAQ: Health Assessment Questionnaire. DAS: The Disease Activity Score.

The Swedish Rheumatology Quality Registry, maintained by the Swedish Society for Rheumatology [27] is a longitudinal registry of incident RA and has been in operation since the mid-1990s. Access to the registry is restricted to clinicians who are treating patients [28]. Researchers and scientific studies need to access the registry and integrate patient records to the above mentioned database. Tens of attributes are included in the SRQ, but not all of them are needed by biomedical researchers. In this research, we modeled and implemented attributes that describe disease duration (temporal) [29], disease activity, and medication. The attributes in the RA registry are entered either by the treating doctor or by the patient, and the RA patients are allowed to assess their disease while at the clinic before meeting with their doctor [30]. An illustration of the list of the attributes from the SRQ and their relationships are shown in Figure 4.

Data curation is a crucial process to integrate data generated in different sites and by different users. We conduct semi-automatic curation procedures based on extract – transform – load (ETL) method for the above sources. To run the ETL we converted all sources into CSV file format and run a nested SQL*Loader (sqlldr) command. SQL*Loader is a utility for data warehouse and described as a high performance database loads [31]. The sqlldr allow the extraction of the CSV file into the Oracle 11 g internal representation and then load the data into the physical database table. The above operation is a mix of a manual command typing as SQL statements and automatic execution of extraction, transformation and load procedures.

Before the ETL take place, the purpose of the curation is to assure the conformance of the each source to the corresponding target schema in the database.

### System implementation

The implementation and deployment of CDC to meet the requirements and listed in requirements matrix (results section), divided into two parts. The first is the creation of the back-end database schemas for each data source and the second part was the implementation of the front-end application and the graphical user interface.

**Figure 5:**
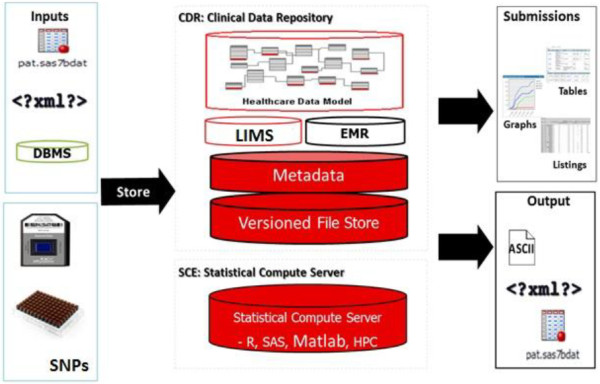
**Clinical development center with its components and information flow ( http://www.oracle.com ).**

CDC uses Oracle11g as back-end. Schemas were created and normalized according to the database design principles and normalizations forms [16]. CDC product of Oracle has the characteristics and functionalities listed in the requirements matrix (see the results section). As a front-end application, the CDC was composed of two sub-systems, CDR- Clinical Data Repository and SCE- Statistical Control Environment. Both CDR and SCE are incorporated with the Oracle Health Care Data Model as shown in Figure 5.

### The clinical data repository

The Clinical Data Repository (CDR) provides a central translational research support system that reduces time and effort required to manage and integrate multiple data sources generated in clinical or research settings. The main features of CDR are as follows:

#### Data source integration

CDR was designed to accommodate and handle different type of file formats and script programs written in SAS® / R-bioconductor or MATLAB®. This gives flexibility for configuring the application to handle datasets produced by laboratory and High-throughput platforms. Data formats are not limited to text files, but they can also be text-based files (e.g., CSV, XML) as well as structured database files. The ability to export data in different formats support interoperability with other software systems and platforms, this is a required feature for data exchange and messaging using XML formats.

CDR users are allowed to import files in many different formats (Excel, comma delimited format, XML and text) to be used for other types of applications or for data exchange between groups and collaborators. The CDR also features version control and data providence mechanisms, which facilitate keeping track of different files and documents that are stored in CDR.

#### Metadata support

Metadata is a dataset that explained the database itself (data about data); metadata is an essential resource in any database management system environment. CDR provides two types of metadata: dataset-level metadata (e.g. date of creation of the database, who create the database, owner of the database, when its published, etc.) and entity-level metadata (this includes information about individual attributes in each database table (e.g. data type, length, range of values, etc.). CDR support creation and documentation of both types of metadata by capturing all data and all changes automatically. Metadata, as a resource, improve the quality of information stored within CDR and also ensured integrity of all data sources. Imported data is validated and reviewed against integrity rules implemented in the schemas prior to transformation and storage in the data repository.

#### Collaboration support and multi-user access to CDR

Inter- and intra-group collaboration is necessary within biomedical research. Providing collaboration and database sharing while maintaining patient data security and privacy is a challenging task. Through a security model that was discussed previously, CDR allows access to all kinds of documents and files that are stored in the database. The CDC security manager increase sharing of the research and clinical database under safe and secure conditions (Principle investigators are able to grant access to the system for co-workers and collaborators.). During requirements elicitation, this was a feature upon which the principal investigator insisted to have.

### Statistical control environment (SCE)

The statistical control environment (SCE) represents automates and tracks the statistical analysis process, providing end-to-end traceability of data, analysis, and assists in generating different types of pre-defined and customizable reports similar to CDR. SCE features meet the requirements of bioinformatics and computational biology. The main features of SCE are as follows:

#### Version control and traceability

CSE provided end-to-end traceability of the computational experiments, and it traced the experiment with all its associated input, programs, and output files. The traceability and version control features are supported with a graphical viewer that helps to visualize dependency relationships among different objects in the experiment (workflow) (also see the results section). It is important for biomedical research that results are re-produceable, and to this end it is necessary that a computational experiment records the version of both data and analysis code, and any parameters used.

Tracing of experiment configurations and version control are supported with a graphical interface that helps visualize dependencies between different objects in the experimental workflow (see also the results section). Integration of version control helps avoid the proliferation of similarly named and marginally different script files that make reproducing old experimental configurations very difficult.

Users of CSE are able to get up-to-date information about the status of different objects stored in CDR at a given point of time.

Retrospective analysis is supported in the CSE to allow reproduction of the data and results from an early analysis. Possibilities to run retrospective analysis is of great importance for the review process and to trace back computational results (see [32]).

### Graphical user-interface

**Figure 6:**
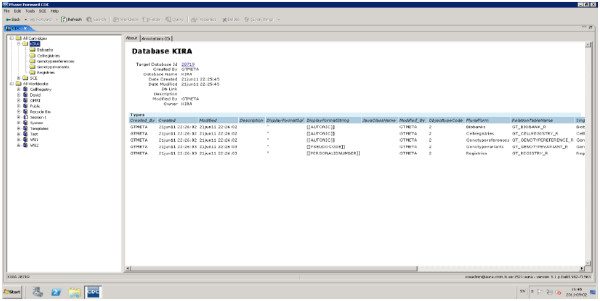
Information architecture and user interface of CDR.

Information architecture (IA) principles aim to create environments with logical structures that help users find answers and complete their tasks [33]. IA is concerned with organization, labeling, navigation, and searching for information [34]. CDC provides the end-user with Windows Explorer-like interface (see Figure 6). This interface has two primary components (panes). The left panes show different objects stored in the CDC under the cartage folders and workbooks. Workbooks consist of folders that are created by the user, such as query folder, programs folders, etc. Each folder has to be created under a workbook, and the types of the folder should be defined when created. The users are only allowed to work with the workbook that was assigned to them by the super-user (PI). The CDC security manager can determine the security settings and access restrictions on the study and folder levels. The cartridge folder is read-only folder, which means that users can explore the content, but are not allowed to perform any writing operations.

User interface for querying different databases is based on a drag-and-drop paradigm. The query builder allows the user to precisely define the input to the query, the attributes, and any kind of query parameter (relational Algebra). Furthermore, users are allowed to join different sources (tables) to maintain a useful query for answering a particular question. The stored queries are editable and able to be saved in different formats, such as SAS and spread sheet applications.

#### Visual query builder

Integrating research and clinical databases for RA cohort, researcher can perform simple queries against a single database (e.g., RA-Biobank) or complex queries, which retrieve results from multiple databases using relationship and primary keys to join tables. They can use logical operators (< , >, =>, <=, Not, like, In) to perform advance query on the database.

A query retrieves a single or group of records for a particular patient or sample. The database aggregates the record(s) to the patient/sample level. In the case of the RA quality registry, the query builder supports retrieval of single patient visits to the clinic. Users can search for a particular SNP, and the result will be extracted from the genotype variants database. To find the risk level of the SNPs, users need to search the genotype reference table, where all SNPs are classified into three categories (Risk, Minor, Major).

The visual query builder generates the corresponding Structured Query Language (Sql) statement, and through the mapping process, the Sql statement is executed on the databases. This reduces the complexity of writing Sql statements for non-technical users.

**Figure 7:**
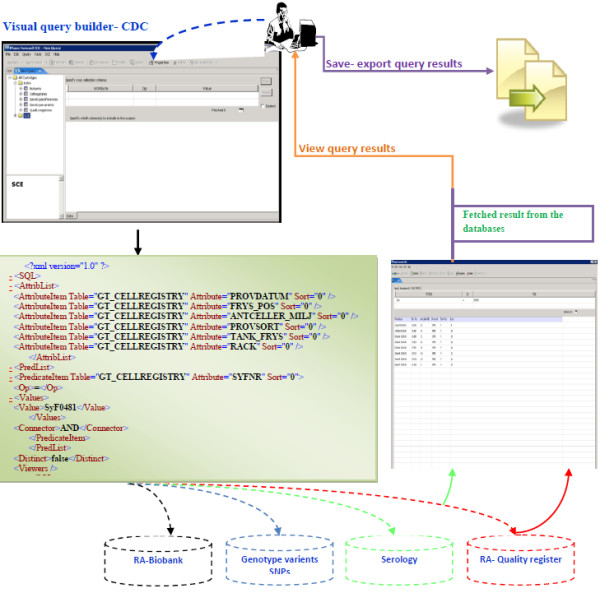
Visual query builder implementation in CDC.

The result is presented to the user in a table format, with columns for each query parameter and rows for each returned value. The user can sort the table by clicking on the column header and selecting “sort type”. In addition, users can remove any of the column headers as long as they are not required. The Sql statement and the results can be saved for future use, or they can be exported to different formats and platforms (The query process is illustrated in Figure 7).

## Results and discussion

### Requirements matrix

In order to facilitate efficient integration and management of workflow, we defined a simple requirements matrix to allow us to evaluate different software solutions and tools. We also provided a detailed list of the tasks from the user’s point of view and then implemented it in CDC.

We compared i2b2, STRIDE and CDC (see section i2b2 and STRIDE: community driven software solutions) and how each of them supports the listed requirements in Table 2.

**Table 2:** List of requirements and a comparison with existing solutions

	**Requirements**	**Description**	**CDC**	**STRIDE**	**i2b2**
R1	Database loading and integration	The platform should provide a visual and easy-to-use user interface to load and transfer data across all studies. The user should be protected from error by having the system validate the source data files before loading them into the database(s).	**√**	**X**	**X**
R2	User-role authentication	System and application level authentication techniques should be supported, the PI wants to grant access to his/her co-workers and collaborator and define specific role and privilege.	**√**	**√**	**X**
R3	Support for bioinformatics work flow developments	Having all the data loaded into the database, the platform should support development of bioinformatics workflow with less scripting effort.	**√**	**X**	**X**
R4	Visual Query Builder	The platform should provide a query builder to easily create and execute queries against the database tables contained in the study.	**√**	**√**	**√**
R5	Data export	The platform should allow exporting query results into different formats, e.g., CSV, spreadsheet	**√**	**√**	**√**
R6	Version control and traceability	The platform should offer version control for all datasets that are stored in the database. Multiple versions of each file are necessary for traceability between inputs and outputs maintained so that the user can view the earlier versions of each file.	**√**	**X**	**X**
R7	Minimal programming effort	The integration of different databases (e.g., biobank, clinical, genotype, etc.) and development of workflow must be easy and require as little programming effort as possible. The platforms should support integration of scripts, e.g., R-script code into the bioinformatics analysis workflow.	**√**	**X**	**X**
R8	Source schema customization and metadata management	The platforms should support creation and documentation of metadata for all data files.	**√**	**√**	**√**
R9	Dashboard display for studies	With a single click on the list of studies, the PI can navigate to different studies running in his/her group and its associated databases.	**√**	**√**	**√**
R10	Installation	The customization and installation effort is minimal.	**√**	**X**	**X**
Total of 10 requirements			**10**	**5**	**4**

Each of the three platforms was developed on specific software architecture. While i2b2 and STRIDE composed of different cells communicating via web service and TCP/IP protocol. CDC is a client server architecture composed of a back-end and a front-end. From our experience the latest is easy to install and maintain and demands less engineering work.

### Authorization, security, and patient’s data protection

Integrating all datasets from the RA quality registry, biobanks, genomics, and cell registry in one data layer creates a security and authorization challenge. The security requirements on patient and research databases are essential and a prerequisite to operate and run a translational medicine platform. A security system based on user roles is essential to ensure that the users can only access information that they are authorized to access. Study level permission is among the best solutions: Scientist ‘X’ is authorized to access the database created for a particular study. PIs can act as super-users to grant access to their collaborators and assistants.

Identifiable patient data cannot be used in research settings. Anonymity, in other words, the removal of personal health information, such as personal identification numbers, names, hospital names, and treating physician names, were removed, and new identification keys were created to facilitate the link between different patient records.

**Figure 8:**
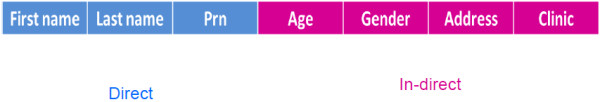
Classification of personal health information (PHI).

According to Health Insurance Portability and Accountability Act (HIPPA) identifiable fields within any EMR (Personal Health Information) are divided into two categories (1) direct attributes which are linked directly to individual or indirect ones such as age, address. See Figure 8.

All attributes in Table 3 (RA-Biobank and SRQ) are removed and replaced with artificial pseudocode generated from the Swedish personal number. This pseudocode will be useful for tracking back the patient, through this process we granted the protection of the individual privacy and allowed the researchers to access clinical data through the platform in a secure way.

**Table 3:** List of PHI per data sources

**Data sources**	**Identifiable attributes**
RA-Biobank	Personnummer - Civic registration number
	Efternamn – last name
	Förnamn- first name
Swedish Rheumatology Quality Register	Personnummer - Civic registration number
	Patientkod: patient registration number in the registry
	Namn: full name
	registrerad_pa_mottagning: clininc address

To assure high level of security, the generation of the pseudocode was performed outside the platform and before database uploads to the server.

The research group obtained ethical permit from the ethical committee at Karolinska Institutet to run the research. Patients' consent form collected and stored in the Rheumatology clinic. Currently there is no support for handling patients consent in the current version of CDC. Handling of the patient’ consent has not been expressed as a requirement when we defined the system functional requirements for this proof of principle study. CDC as a software platform is able to expand its capabilities and services to allow the storage of the patient’ consent forms.

The de-identification process represents the first security layer in this system, and this complemented with more layers to protect the privacy and also the intellectual property rights and ownership of the database. In the rest of the section we are going to discuss more about the security protocol implemented in the system.

In addition to the above, advanced encryption techniques were embedded in CDC to encrypt user names and passwords stored in different system directories. Users granted the necessary credentials are able to access the system either inside the CMM firewall at Karolinska University Hospital or as remote users outside CMM via virtual private network (VPN).

**Figure 9:**
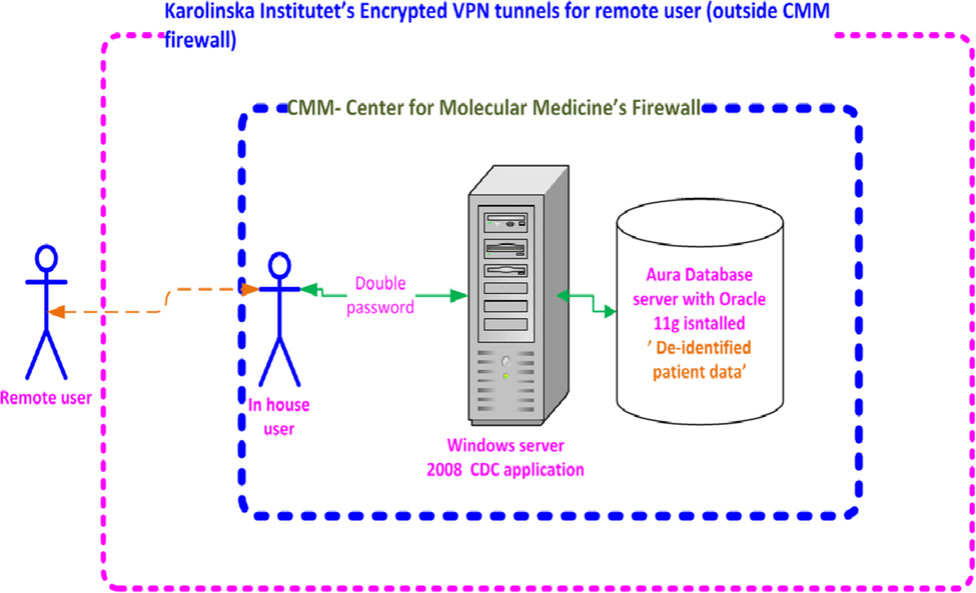
Security layers of the system.

As a good practice, the database server was isolated from the application server, which has both advantages and disadvantages and has been debated within server consolidation and multi-tier-architecture. The security architecture and setup illustrated in Figure 9.

The system was installed on a Windows® 2008 server and accessible through remote access desktop connection, where users needed to have access to the Remote Access Desktop (RAD) provided by the system administrator. This represents the first level credential prior to accessing the CDC. The second password was provided by the PIs to their team members or collaborators, and the PI is able to specify the duration and validate the credential given to specific users. All tasks executed by the users were tracked and logged. Operations, such as deleting a folder or wordbook, were only possible if the user was granted the required permission from the group leader.

### System usability and test cases

To demonstrate CDC usability, we selected and presented two scenarios (test cases). The two scenarios shows the real need for data integration and also potential types of queries that can run across multiple data sources.

### Scenario I: biomedical research: cross-sources search and query

Ease of use and query generation are important features that are required by end-users with less experience when constructing query commands. Through the CDC visual query builder, biomedical researchers are able to compose and run a query across data sources. CDC is windows based and the graphical user interface (GUI) uses the same paradigm of interaction as all GUI. The biomedical researcher familiar with windows applications is able to run the queries by drag and drop techniques and they don’t need to write a single code statement or script. We provided a user manual that help and explain necessary steps to run a query and visualize the results see Figure 7.

The following query plan shows an example of how a sample was selected based on HLA (Human Leukocyte Antigen) type and a precondition of having a number of cells greater than 50.60^6^ and SNP rs2064476 = AG and GG:

#### Select

*RS2064476*,GTA_71.

*SYFNR*, GTA_71.

*ANTCELLER_MILJ*,GTA_71.

*FRYS_POS*,GTA_75.

*HLA_TYPE*,GTA_71.

*PROVSORT*,GTA_75.

CCP_SERUM

#### From

*GT_GENOTYPEVARIANT GTA_75*, GT_CELLREGISTRY GTA_71

#### Where

**HLA_TYPE**  =  ('*01/*04','*03/*04','*04','*04/*04','*04/*07','*04/*08','*04/*09','*04/*10','*04/*11','*04/*12','*04/*13','*04/*14','*04/*15','*04/*16')

AND

GTA_71.**ANTCELLER_MILJ** >= '50'

AND

GTA_75.**RS2064476** in ('AG','GG'))

The XML schema for the above query is illustrated in standard XML format (XML_HLA_query.xml) and the application schema (XML_HLA_query.xsd ) are available in appendix 1

The result of the above query plan illustrated in Table 4:

**Table 4:** Result of query implemented in CDC visual query builder

**Rs2064476**	**Syfnr**	**Number of cell in million**	**Freezer position**	**HLA type**	**Sample type**	**Ccp serum**
AG	SyF0420	53	01:G09	*04/*15	SFMC	938.9
AG	SyF0420	53	01:G10	*04/*15	SFMC	938.9
AG	SyF0420	53	01:H01	*04/*15	SFMC	938.9
AG	SyF0420	53	01:H02	*04/*15	SFMC	938.9
AG	SyF0420	53	01:H03	*04/*15	SFMC	938.9
AG	SyF0420	53	01:H04	*04/*15	SFMC	938.9
AG	SyF0420	53	01:H05	*04/*15	SFMC	938.9

### Scenario II: bioinformatics application: for comparing antibodies vs. SNP

The CDC statistical development center allows the development of programs written in different programming/scripting   languages   (SAS®,  S-PLUS®,  R-  bioconductor, MATLAB®, or PFS®).

Based on CDC workflow capabilities, an analytical pipeline was defined to report a very detailed and specific analysis. This pipeline was incorporated in two parts: the first part queries the patients of interest, and the second part runs an R-script in the SCE to identify genotypes (SNPs) of interest.

The analysis of the query’s output (QO) was performed by using a R-script, the R-script performs the following tasks sequentially:

1) Basic statistical summary of QO (such as prevalence, mean, standard deviation);

2) Classification of the SNPs as major, minor and/or risk allele comparing the classification with the provided (if provided) QO;

3) Computing the odds ratio for the different SNPs and the different antibodies; and

4) Plotting, for each antigen, those SNPs that are statistically associated (after false-discovery rate correction) as risk alleles.

The above bioinformatics study aim is to investigate how non-DRB1 genetic polymorphism may contribute to the development of certain serological types of RA in individuals with defined DRB1 genotypes.

### Improve research data management & collaboration among groups

In addition to the data integration possibilities, the implementation of CDC improves research data management. Firstly, CDC provide flexible and scalable infrastructure, which will allow management of in-house research databases (Biobanks, serology, and genetics). Secondly, it increases collaboration across different research groups within CMM working in different therapeutic areas. Importantly, the platform will automate exchange of diverse data among research groups. Thirdly, it enabled access to the clinical data stored in the quality of care registry at the clinic through an intuitive and usable system.

We also summarized the road-blocks in scaling-up the solutions to other diseases as the following:

### Absence of data management methodology

One of the main obstacles to running a software solution for data integration is the lack of a methodological and systematic approach to capture, store, and analyze biomedical and clinical data. Currently, individual researchers use their own ad-hoc systems for storing raw data and results. The software solution needs to provide an efficient and systematic way for handling a large number of databases. Verification of different data sources, which may be in different formats and media, can be an additional task in the process (tractability, version control, and quality control). Verification and version control of data files have a direct impact on bioinformatics and upstream analysis. The lack of an appropriate version control system and quality control procedures reduces the usability of the available data and increases the time required for data curation and preprocessing procedures ([35-37]).

### Data linkages and quality of information flow

Biomedical data are captured and stored by individuals (biobankers, biologists, research nurses, lab technicians). Without proper indexing and metadata on datasets and attributes, this will affect data access, integration, and linkages between different data sources for a particular patient or sample [38]. Ideally, since there are a number of data sources available, it is important to note that the data sources should be linked (using join/primary keys) and indexed in a way that makes such data retrieval possible and easy. To obtain maximum value from the data, structured and accessible metadata is required. Proper indexing and linking of different data sources will contribute to the quality of information flow.

### Absence of metadata

There are number of potential problems associated with the definition of individual attributes (fields). For example, only the data owner (researcher) can interpret the naming convention, which, in addition to the lack of metadata, will reduce the utilization and reusability of the research data by collaborators. This is one of the major difficulties that we faced during the conceptual and physical design of the schemas. Ad-hoc naming and codes affected the quality and re-usability of the datasets. This is due to the fact that no data dictionary is in place, and researchers have less time to pick appropriate names for attributes from available ontologies or thesauruses. One attribute has more than one name among the different sources. Table 5 shows examples of the same attribute has different name in different database sources, which causes a semantic problem during integration.

**Table 5:** **Different naming used for same attribute in different database source**s

**Genotype variant**	**Cell registry**	**RA- Biobank**
Project number	SyRnr	Patienter::Pat.nr. (patient number)
Personnr. + Personnr2 (Date of birth + Personal identification 4 digits number)		Patienter::Personnummer + Patienter::Pers.dat (Date of birth + Personal identification 4 digits number)
Gender		Patienter::Man_Kv
	Provdatum (sample date)	Provdat (sample date)
	Provsort (sample type)	Provtyp (sample type)
Namn (Name)		Patienter::Efternamn (last name) + Patienter::Förnamn (first name)

The willingness of individual PIs and researchers to share their data and collaborate is the cornerstone of a successful and effective information sharing system. In addition, attributes, and metadata should be defined and agreed upon; this data set can be regarded as common vocabulary, which is essential for comparing the analysis results in different experiments and laboratories.

### Individual’s genomics information

Protecting individual’s genomics information become a challenge and debated in ([39-43]). There is no technical solution to protect re-identification of individuals, as has been proved in [44] the availability of summary level allele frequency for two matched sample groups and the genome profile of one of the participants (subject of interest), will make it easy to re-identification of individuals (subject/ person). The current trends in genomics database try to hide some of the genotype / phenotype information to reduce the risk of re-identification of individual.

## Conclusions

In this research we addressed the challenges facing biomedical and clinical researcher in a translational research environment.

The implementation of CDC as a translational informatics platform integrates clinical and biomedical databases for Rheumatoid Arthritis. We developed a requirements matrix that captures the requirements of end users. We matched the matrix with available technologies, and we compared CDC against community-driven translational platforms (i2b2 and STRIDE). Then, we implemented CDC, the architecture of which provided the requirements for the two classes of end users, biomedical researchers and bioinformaticians. Through CDC, biomedical investigators are able to store, access, and retrieve sample databases from the biobanks and integrate it with the genetics and serology data for the cohort under study. Additionally, researchers are able to re-use clinical data from the quality of care registry and run more complex queries across different sources (genetics, serology, medication, diagnosis and sample related parameters). The architecture and user interface of CDC reduce the time spend by researchers to preselect biological samples (from the RA biobanks) based on clinical parameters (diseases activity, medication) in a systematic manner. The CDC, through its visual query builder, allows the preparation of the research material based on both the availability of the samples, suitable genotype, or serology status, and on disease parameters from the SRQ.

Bioinformaticians are able to create a workflow and run computation jobs based on the stored databases. We demonstrated the use of the workflow and version control capabilities of CDC with examples of comparing antibodies vs. SNPs.

During the development we experienced difficulties to collect and curate the dataset coming from different sources. Data curation is a time consuming task and a crucial process to make sure that source data files contains all attributes with the appropriate data structure.

One of the main challenges is to provide a secure and trustable system for biomedical researcher. We considered all security and ethical regulations pertaining to patient data. To assure the utilization of the system, we arranged training sessions to the researchers; we explained and demonstrated the functionalities by real example and listen to their question.

We used Rheumatoid Arthritis as a test case since we have ample data readily available.

There are many opportunities for future development, and we envision that the implementation of CDC at the Rheumatology Unit will drastically evolve from RA to other diseases and research groups in inflammatory and cardiovascular diseases.

## Abbreviations

CDC: Clinical Development Center; CDR: Clinical Data Repository; EMR: Electronic Medical Record; ETL: Extract-Transform-Load; HL7: Health Level Seven; HGVS: Human Genome Variation Society; ICD: The International Classification Of Diseases; OEM: Ontology Engineering Methodology; PHI: Personal Health Information; RA: Rheumatoid Arthritis; SCE: Statistical Control Environment; SNOMED: Systematized Nomenclature Of Medicine Clinical Terms; SNP: Single-Nucleotide Polymorphism; SRQ: Swedish Rheumatology Quality Register; UML: Unified Modeling Language

## Competing interests

The authors declare no competing of interests.

## Authors’ contributions

Conceived and designed the study: IA LK VM JT. Performed the biological experiments: OS. Performed the bioinformatics study: DGC. Wrote the paper: IA DGC VM JT: SL provided access to the clinical data from SRQ. All authors read and proofed the final manuscript.
